# MRI-Based Evaluation of PRP Therapy in Knee Osteoarthritis: WORMS and Synovial Changes at 6 Months

**DOI:** 10.3390/jcm14186408

**Published:** 2025-09-11

**Authors:** Takanori Wakayama, Yoshitomo Saita, Sayuri Uchino, Yohei Kobayashi, Hirofumi Nishio, Shin Fukusato, Yasumasa Momoi, Hiroshi Ikeda, Kazuo Kaneko, Muneaki Ishijima

**Affiliations:** 1 Department of Orthopaedics, Juntendo University, 2-1-1 Hongo, Tokyo 113-8431, Japan; twakaya@juntendo.ac.jp (T.W.); s-uchino@juntendo.ac.jp (S.U.); yhkobaya@juntendo.ac.jp (Y.K.); h-nishio@juntendo.ac.jp (H.N.); s-fukusato@juntendo.ac.jp (S.F.); y-momoi@juntendo.ac.jp (Y.M.); hi-ikeda@juntendo.ac.jp (H.I.); k-kaneko@juntendo.ac.jp (K.K.); ishijima@juntendo.ac.jp (M.I.); 2 Department of Regenerative Medicine for Locomotive Organ, Juntendo University, 2-1-1 Hongo, Tokyo 113-8431, Japan; 3 Faculty of Health Science, Juntendo University, 2-1-1 Hongo, Tokyo 113-8431, Japan

**Keywords:** platelet-rich plasma, pain, knee osteoarthritis, regenerative therapy, articular cartilage repair

## Abstract

**Objective:** Platelet-rich plasma (PRP) therapy has become a popular treatment for knee osteoarthritis. We aimed to determine the outcomes of knee osteoarthritis patients following PRP therapy using magnetic resonance imaging (MRI) findings and patient-reported outcome measures (PROMs). **Design:** In this retrospective observational cohort study, we enrolled 161 patients (221 knees) with varus knee osteoarthritis who received multiple PRP injections at our hospital from June 2017 to June 2019. Patients underwent whole-body MRI before and 6 months after treatment. Whole-organ MRI score (WORMS) cartilage integrity and synovial fluid volume were assessed for the medial femorotibial (MFTJ), lateral femorotibial (LFTJ), and patellofemoral joints (PFJ). Pain visual analog scale and Knee Injury and Osteoarthritis Outcome scores were used as PROMs. In addition, a historical control group of 30 patients with medial knee osteoarthritis who did not receive intra-articular injections was evaluated by MRI over the same period for comparison. **Results:** After 6 months of PRP therapy, the mean WORMS cartilage score of the LFTJ and PFJ and the total WORMS cartilage score for all three joints improved significantly, and synovial fluid volume reduced significantly. Moreover, a reduction in synovial fluid volume correlated with improvements in several KOOS subscales but not with VAS, which may explain the lack of association with responder status. These results suggest that synovial fluid reduction reflects functional improvement but is not a direct surrogate for pain relief. In addition, the change score of WORMS PFJ cartilage correlated positively with clinical outcomes in responders. By contrast, in the control group, no compartment demonstrated improvement in WORMS cartilage scores, and several compartments showed a trend toward deterioration. **Conclusions:** In this retrospective observational study, PRP therapy was associated with improvements in WORMS cartilage integrity scores and reductions in synovial fluid volume, with partial correlations to patient-reported outcomes. The inclusion of a historical control group strengthens the interpretation of these findings, although definitive conclusions cannot be drawn. Further randomized controlled trials are needed to confirm these preliminary observations.

## 1. Introduction

The incidence of knee osteoarthritis (OA) is increasing in aging societies and is a major public health concern worldwide. Disability caused by degenerative joint diseases may result in the need for long-term care. Symptomatic knee OA occurs in approximately 10% of men and 13% of women aged 60 years or older [1]. Both conservative and surgical treatments have been found to improve many patients’ symptoms and daily activities. However, a proportion of patients do not respond to any currently available conservative therapies. Therefore, there is an urgent need for the development and assessment of novel disease-modifying therapies.

Orthobiologics are new biological therapies for joint and musculoskeletal diseases [2,3] and are aimed at improving disease symptoms as well as accelerating the restoration of damaged tissue, such as articular cartilage. Several systematic reviews and meta-analyses of Level I and II studies have demonstrated the safety and clinical benefit of platelet-rich plasma (PRP) for knee OA [4]. For example, several studies have shown that PRP administration has a positive effect on chondrocyte differentiation and proliferation and extracellular matrix synthesis [5,6]. According to the epiligament theory, the epiligament supplies cells and vascularization, essential for ligament healing. In this context, PRP has also been reported to enhance ligament healing in preclinical models, although clinical evidence remains limited [7]. In a systematic review, Boffa et al. described that in animal OA models, intra-articular PRP injections induce disease-modifying effects at both the cartilage and synovial levels [8]. However, whether PRP therapy halts or reverses the degeneration of articular cartilage in humans remains unknown. Therefore, we investigated whether PRP injection for knee OA improves objective magnetic resonance imaging (MRI) findings, such as the cartilage score of the whole-organ MRI score (WORMS) and synovial fluid volume [9]. Additionally, we explored the link between patient-reported outcome measures (PROMs) and MRI outcomes. We hypothesized that 1) PRP injection for knee OA would improve the WORMS cartilage score and decrease synovial fluid volume, and 2) the improved WORMS cartilage score following PRP therapy would be associated with improved PROMs.

## 2. Materials and Methods

### 2.1. Patients

This study involved a consecutive series of PRP injections in 221 knees of 161 patients performed at our institution from June 2017 to June 2019. Both 3.0 Tesla MRI and PROMs data before and 6 months after PRP injections were available for all patients. The indications for PRP therapy were knee OA regardless of joint deformity severity and the presence of continuous knee pain for at least 1 year despite the use of other conservative treatments, such as nonsteroidal anti-inflammatory drugs (NSAIDs) and intra-articular hyaluronic acid injection. The exclusion criteria were systemic inflammatory diseases (e.g., rheumatoid arthritis), active infectious diseases, platelet disorders or diseases, immunosuppression, a history of cancer, use of NSAIDs in the 2 days before the injection, and corticosteroid injection in the knee in the 3 weeks before the injection. This retrospective observational cohort study was approved by the ethics committee of our institution (#20-062). Informed consent was obtained from all participating patients.

A control group was established to observe changes in knee cartilage and synovial fluid over a 6-month period. Patients were retrospectively identified from electronic medical records based on the following criteria: individuals diagnosed with medial-type knee osteoarthritis who underwent MRI at both the initial visit and at 6 months and did not receive any intra-articular injections of PRP, hyaluronic acid, or corticosteroids during the observation period. A total of 30 patients meeting these criteria were included as the control group to determine whether the changes observed in the PRP group reflected the natural course of the disease or the effects of treatment. This control group was evaluated only by MRI findings, and clinical outcome measures such as the VAS or KOOS were not available.

### 2.2. Radiological Analyses

**Figure 1:**
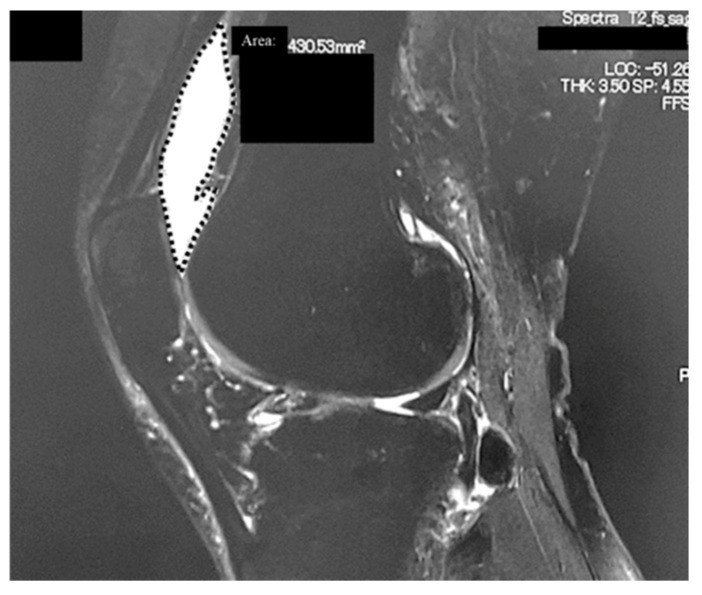
Analysis of the area of synovial fluid in the suprapatellar synovial bursa.

A standing anteroposterior radiograph was obtained to evaluate the grade of knee OA using the Kellgren–Lawrence (KL) classification system [10]. Whole-body MRI scans were acquired using a 3.0 Tesla scanner (MAGNETOM Spectra; Siemens Healthineers, Erlangen, Germany or Intera Achieva 3.0T TX; Philips, Amsterdam, The Netherlands). Images were analyzed by two orthopedists (WK and SU) who were blinded to the clinical outcomes of the patients. Semi-quantitative MRI-based scoring was performed using the WORMS [9]. Of the 14 features incorporated into the WORMS, we analyzed the articular cartilage integrity and subarticular bone marrow abnormality (BMA) scores. Cartilage signal and morphology were scored as follows: 0.0 = normal thickness and signal on T2-weighted images; 1.0 = normal thickness but increased signal on T2-weighted images; 2.0 = partial-thickness focal defect < 1 cm at the widest point; 2.5 = full-thickness focal defect < 1 cm at the widest point; 3.0 = multiple areas of partial-thickness (grade 2.0) defects intermixed with areas of normal thickness or a grade 2.0 defect wider than 1 cm but encompassing <75% of the region; 4.0 = diffuse (≥75% of the region) partial-thickness loss; 5.0 = multiple areas of full-thickness loss (grade 2.5) or a grade 2.5 lesion wider than 1 cm but encompassing <75% of the region; and 6.0 = diffuse (≥75% of the region) full-thickness loss. The maximum cartilage scores for the medial femorotibial joint (MFTJ), lateral femorotibial joint (LFTJ), patellofemoral joint (PFJ), and entire knee were 30, 30, 24, and 84, respectively. The interobserver agreement of the WORMS was high (most intraclass correlation coefficients were >0.80) [9]. We analyzed three articular surface areas: the PFJ, MFTJ, and LFTJ. For the analysis of the area of synovial fluid in the suprapatellar synovial bursa, we set the region of interest in the sagittal section of the fat-suppressed T2-weighted fast spin-echo sequence. The high T2-intensity area within the suprapatellar bursa was quantified using a picture archiving and communication system viewer/workstation (Synapse, Fujifilm Corporation, Tokyo, Japan; Figure 1).

The regions of interest were set at the sagittal section of fat-suppressed T2-weighted fast spin-echo sequences. The T2 high-intensity area within the suprapatellar bursa was measured using the PACS viewer/workstation.

### 2.3. Clinical Outcomes

Clinical outcomes were assessed by changes in the visual analog scale (VAS) score for pain and the Knee Injury and Osteoarthritis Outcome Score (KOOS), a self-administered PROM [11]. The items of the KOOS are graded on a five-point Likert scale from 0 to 4 and encompass the following five subscales: pain, symptoms, activities of daily living, sport and recreation, and knee-related quality of life [11]. PRP effectiveness was determined using the Outcome Measures in Rheumatology responder criteria (established by the Osteoarthritis Research Society International [OMERACT-OARSI]) [12], which are based on a combination of absolute and relative changes in pain, function, and global patient assessment. The patients were classified as responders if one of the following two criteria was fulfilled: (1) high improvement in pain: ≥50% improvement + absolute change of ≥20 in pain, or (2) improvement in at least two of the three following items: ≥20% improvement + absolute change of ≥10 in pain, ≥20% improvement + absolute change of ≥10 in function, or ≥20% improvement + absolute change of ≥10 in the patient global assessment of disease activity.

### 2.4. PRP Preparation and Injection

The protocol and ethics of PRP therapy were certified by a special committee for regenerative medicine according to the local act regulating the safety of regenerative medicine (approval number PB3150023). The PRP preparation was obtained via single centrifugation of whole blood using the MyCells autologous platelet preparation system (Kaylight Ltd., Ramat HaSharon, Israel). Briefly, 22 mL of whole blood was aspirated into two sets of MyCells kit syringes containing 1 mL of anticoagulant dextrose solution A and separation gel. Next, the samples were centrifuged for 7 min at 2000× *g* at 21 °C to 25 °C. After aspirating the supernatant platelet-poor plasma, 2.0–2.5 mL of residual plasma was pipetted to remove platelets from the surface of the separation gel. The filter column was then inserted into the separation syringe to remove the debris and filtered PRP. The final volume of liquid PRP product was 4–5 mL. The mean platelet counts of the PRP and whole blood across all patients were 46.9 ± 18.0 × 10^4^/μL and 22.2 ± 6.6 × 10^4^/μL, respectively. The mean white blood cell count of the PRP and whole blood were 1807 ± 1185/μL and 4477 ± 176/μL, and the mean red blood cell count of the PRP and whole blood were 3.3 ± 13.3 × 10^4^/μL and 455.2 ± 68.4 × 10^4^/μL, respectively. Therefore, the mean platelet concentration was 2.14-fold (±0.87) greater than that of whole blood, and the mean white blood cell concentration was 0.36-fold (±0.38) lower than that of whole blood. The mean platelet recovery rate of this protocol was 44.6 ± 17.1%. This was classified as P2-Bβ PRP (leukocyte-poor PRP) according to the platelet, activation, white blood cells classification system [13]. Nonactivated PRP was injected into the suprapatellar bursa via the lateral suprapatellar approach using a 21-gauge needle within 30 min of the blood draw at 21 °C. If aspiration of the joint fluid was possible, this was performed before the PRP injection. As per our standard protocol, leukocyte-poor PRP (4–5 mL), comprising three injections, was administered every 4 weeks. In addition, all patients were instructed in a standardized home exercise program developed by physical therapists. The program included straight leg raise training, hip abduction training, and hip adduction training, aiming to improve periarticular muscle strength and joint stability. However, patient adherence to the program was not systematically assessed.

### 2.5. Statistical Analyses

Changes between pretreatment and posttreatment VAS, KOOS, and MRI scores were analyzed using the Wilcoxon test because the scores were not normally distributed, as confirmed by the Shapiro–Wilk test (*p* < 0.05). Logistic regression was performed using the OMERACT-OARSI responder criteria as a binary outcome to determine whether the changes in objective MRI measures were related to the effectiveness of PRP therapy. All statistical analyses were performed using SPSS version 20.0 (IBM Corp., Armonk, NY, USA). All *p*-values were two-sided, and *p* < 0.05 was considered statistically significant.

## 3. Results

### 3.1. Efficacy of PRP Therapy

Nearly half (49.3%) of all knees had KL grade 4 OA, 26.2% had KL grade 3 OA, and 24.4% had KL grade 2 OA (Table 1). Six months after the PRP injection, there was a significant improvement in the VAS and KOOS subscale scores (Table 2). The responder rate of patients who met the OMERACT-OARSI responder criteria at 6 months was 61%. Baseline characteristics of age, sex, body mass index, KL grade, and MRI score did not differ significantly between responder and non-responder groups. However, the baseline VAS and KOOS were significantly lower in the responder group than in the non-responder group. The baseline WORMS did not differ between the responder and non-responder groups (Table A1, Appendix A).

### 3.2. MRI Analysis

Table 3 shows the results of the WORMS cartilage score by KL grade. The mean WORMS cartilage score for the MFTJ was 17.9 ± 8.6 at the initiation of PRP therapy, and no improvement was observed 6 months after PRP (18.0 ± 8.9; Table 3). Conversely, the mean WORMS cartilage score for the LFTJ (from 4.7 ± 6.3 to 4.3 ± 6.2; *p* < 0.001) and PFJ (from 7.0 ± 6.1 to 6.8 ± 5.9; *p* = 0.048) improved significantly. The total WORMS cartilage score (i.e., the sum of the scores for the MFTJ, LFTJ, and PFJ) also improved significantly from 29.5 ± 16.9 to 29.0 ± 16.8 (*p* = 0.021). The mean WORMS BMA score did not improve following PRP therapy. The area of synovial fluid in the sagittal section of the suprapatellar pouch decreased significantly from 2.1 ± 1.8 to 1.7 ± 1.6 cm^2^ (*p* < 0.001).

As a control group, MRI analyses were performed in 30 patients with medial knee osteoarthritis (mean age, 67.5 ± 10.7 years) who were observed for six months without receiving any intra-articular injections, including PRP. The control group consisted of 19 patients with KL grade 2, 6 patients with KL grade 3, and 5 patients with KL grade 4. Overall, cartilage scores tended to deteriorate in the MFTJ and PFJ compartments (Table 4). When stratified by KL grade, patients with KL2 showed a significant worsening of the MFTJ cartilage score over six months (3.3 ± 3.3 → 3.6 ± 3.6, *p* = 0.021). In contrast to PRP, no compartment in the control group demonstrated improvement in WORMS cartilage scores, and synovial fluid volume did not decrease. The VAS score was significantly correlated with the WORMS of the MFTJ (*r* = 0.21, *p* = 0.001) and PFJ (*r* = 0.16, *p* = 0.01) and the WORMS BMA score (*r* = 0.15, *p* = 0.02) but not with the area of synovial fluid at baseline (Table 5). The WORMS of the LFTJ showed a trend correlation with the VAS score (*r* = 0.13, *p* = 0.051).

### 3.3. Association Between PROMs and MRI Findings

Changes in MRI findings were compared between responders and non-responders. In responders, the WORMS cartilage scores for the LFTJ, PFJ, and total areas improved significantly (*p* < 0.001, *p* = 0.001, and *p* = 0.001, respectively; Table 6), whereas the WORMS cartilage score did not improve in non-responders. Although the area of synovial fluid showed limited change in responders (*p* = 0.052), non-responders exhibited a significant reduction (*p* < 0.0001).

Furthermore, when analyzed across the entire cohort, correlation analyses revealed that the reduction in synovial fluid volume was not associated with changes in VAS. In contrast, significant positive correlations were observed between synovial fluid reduction and improvements in several KOOS subscales, including pain (r = 0.16, *p* = 0.018), symptoms (r = 0.17, *p* = 0.013), activities of daily living (r = 0.18, *p* = 0.007), and quality of life (r = 0.15, *p* = 0.025), but not sports/recreation (r = 0.12, *p* = 0.074) (Table 7). These findings suggest that reductions in synovial fluid volume were related to improvements in most KOOS domains, despite the lack of association with VAS.

In the multivariable logistic regression analysis (Table 8), responders showed significant improvement in WORMS cartilage scores for the PFJ (odds ratio, 1.45; 95% confidence interval, 1.09–1.94; *p* = 0.01).

## 4. Discussion

The main finding of this study is that WORMS cartilage scores improved significantly for the LFTJ and PFJ, but not the MFTJ, and the area of synovial fluid decreased significantly 6 months after the PRP injection. These observations suggested that PRP induces both disease-modifying and anti-inflammatory effects. PROMs improved significantly 6 months after the PRP injection; moreover, in responders, the WORMS cartilage score for the PFJ improved significantly and was associated with symptom improvement.

Several systematic reviews and meta-analyses have demonstrated the clinical benefit of PRP for knee OA [4]. However, most of the large PRP therapy studies for knee OA only evaluated PROMs [14,15,16]. MRI analysis is useful for objective examination of clinical improvement following PRP treatment. However, previous investigations into changes in articular cartilage condition following PRP injection have concluded that articular cartilage does not improve with PRP treatment [17,18]. The discrepancy between our and previous studies may be because previous studies focused only on the most severely damaged area in varus knee OA, such as the MFTJ. In a large randomized controlled trial (RCT) conducted by Bennell et al., only the medial tibial cartilage, a high-bearing site, was evaluated using MRI. We similarly observed a lack of improvement in the WORMS cartilage score for the MFTJ, whereas we found significant improvement in the scores for the LFTJ and the PFJ, which are low-bearing sites. In this study, we referred to “mechanically stable” knees as those without clinical signs of dynamic instability, such as varus thrust during gait, and with relatively preserved joint alignment. In the context of varus knee osteoarthritis, the MFTJ typically bears the greatest load and shows more advanced cartilage degeneration, whereas the LFTJ and the PFJ are often subjected to lower mechanical stress and maintain better structural integrity. The PFJ, in particular, is considered more biomechanically stable in early-stage knee OA. This relative mechanical stability may provide a more favorable environment for biologic therapies such as PRP to exert their potential disease-modifying effects. The structural improvements we observed in the LFTJ and PFJ support this hypothesis.

In vitro findings have demonstrated that PRP positively influences not only catabolic and inflammatory processes but also structural features at the joint cell level [19]. A double-blind RCT conducted by Raeissadat et al. showed that patellofemoral cartilage volume, as measured by MRI, improved significantly 8 months after PRP treatment for knee OA [20]. Consistent with this trial, we observed an improvement in the WORMS cartilage score for the PFJ (Table 3).

Dong et al. compared the cartilage status of the medial compartment following high tibial osteotomy among the interventions of PRP, hyaluronic acid, and normal saline using MRI and arthroscopy and showed that the cartilage condition of the PRP group improved significantly 12 months postoperatively [21]. Taken together, these findings suggest that in humans, PRP may possess disease-modifying abilities, such as regeneration of articular cartilage, especially in low-weight-bearing sites in those with mechanically stable knee joints. In our study, the WORMS cartilage score for the LFTJ in the varus knee joint improved significantly following PRP therapy (Table 3). Therefore, the disease-modifying effect of PRP therapy may be enhanced by combining it with other knee joint-stabilization therapies, such as physiotherapy and around-knee osteotomy. In this study, all patients were also prescribed a home-based exercise program designed by physical therapists. The inclusion of exercise therapy represents a significant co-intervention that may have contributed to improvements in both symptoms and joint stability. As established in the 2019 OARSI guidelines [22], exercise is a core treatment for knee osteoarthritis, with well-documented effects on pain and function. Furthermore, a recent scoping review by Oka et al. reported that combining regenerative medicine with exercise therapy significantly enhances clinical outcomes in patients with knee OA [23]. Therefore, it is plausible that the observed improvements in PROMs, WORMS cartilage integrity scores, and synovial fluid volume may be partially or wholly attributable to the synergistic effects of PRP and exercise therapy. This potential confounding factor should be considered when interpreting the results of this study.

In this study, we compared MRI changes between patients with Knee OA who received intra-articular PRP injections and those who were observed without such treatment. Interestingly, whereas most compartments in the PRP group remained stable and some even demonstrated improvement (Table 3), no compartment in the control group showed improvement (Table 4), and several exhibited a trend toward deterioration. While the chondroprotective effects of PRP have been demonstrated in cell and animal models, they have not previously been clearly shown in patients with Knee OA. The present findings provide important evidence suggesting that intra-articular PRP injections may exert a chondroprotective effect in Knee OA. Future prospective, well-designed studies are warranted to confirm these results.

The cause of pain in patients with knee OA is complex [24]. We found that the WORMS cartilage scores for the MFTJ and PFJ and the WORMS BMA score correlated with the VAS score (Table 5). This suggests that pain in patients with knee OA is caused not only by severely damaged sites (assessed radiographically) but also by sites with mild to moderate cartilage damage and subchondral BMA. Interestingly, the improvement in WORMS cartilage score for the PFJ was associated with improved symptoms, which suggests that symptom improvement following PRP therapy is not necessarily attributed to cartilage repair in areas with the greatest damage. Notably, the baseline VAS and KOOS were significantly worse in the responder group compared to the non-responder group. This may indicate that patients with more severe symptoms had greater capacity for improvement, a pattern commonly observed in clinical research. Such differences could reflect regression to the mean or greater responsiveness to intervention among those with higher baseline symptom burden. This observation suggests that baseline symptom severity may influence treatment response and could be considered when selecting candidates for PRP therapy in future studies. In this study, symptom improvement was analyzed by classifying patients as responders. However, since there were significant differences in the baseline VAS and KOOS between the responder and non-responder groups prior to treatment, the possibility of patient selection bias cannot be excluded. Therefore, further research is necessary to explore the relationship between changes in articular cartilage and PROMs.

Preclinical evidence has demonstrated that PRP reduces inflammatory reactions caused by OA: PRP injections decrease synovial fluid concentrations of tumor necrosis factor-α (TNF-α) and interleukin-1 beta (IL-1β) [8]. In our study, the area of synovial fluid significantly decreased following PRP therapy. Interestingly, the reduction in synovial fluid volume correlated with improvements in most KOOS subscales but not with VAS. This discrepancy may explain why synovial fluid reduction was not associated with responder status, which is primarily determined by VAS and KOOS-ADL. Our findings suggest that while decreased synovial fluid volume may reflect improvements in functional domains of knee OA, pain perception may be more closely linked to biochemical or inflammatory factors within the synovial fluid rather than its absolute volume. Future studies combining biomarker analyses with imaging may clarify this complex relationship between fluid dynamics and clinical outcomes. However, improvement in the area of synovial fluid was not correlated with clinical outcomes (Table 5 and Table 7). In another RCT, Chu et al. reported that the levels of TNF-α and IL-1β in synovial fluid were significantly lower 6 months after PRP injection than at baseline [25]. Therefore, in terms of pain reduction, inflammatory cytokine levels in synovial fluid may be more important than synovial fluid volume. It is conceivable that the reduction in synovial fluid volume is attributed to changes in synovial fluid biomarkers and anti-inflammatory effects.

In addition, although demographic factors such as age and BMI were collected in this study, the relatively small sample size limited our ability to reliably evaluate their potential influence on imaging and clinical outcomes. Given that BMI in particular may affect joint cartilage loading in areas of greatest stress, future studies with larger cohorts are warranted to clarify these associations.

Furthermore, the retrospective and non-controlled nature of this study limits the ability to draw definitive causal inferences regarding the effect of PRP therapy. Although improvements in MRI and PROMs were observed, it remains possible that other factors—such as natural disease fluctuation, concomitant treatments (e.g., exercise therapy), or regression to the mean—may have contributed to the observed changes. Therefore, these findings should be interpreted with caution and viewed as hypothesis-generating rather than confirmatory.

This study has several important limitations. Most notably, it employed a single-arm, retrospective observational design without a control group. As a result, the improvements observed in MRI findings and clinical outcomes cannot be definitively attributed to the PRP injections alone. The absence of a comparison arm makes it impossible to exclude alternative explanations such as placebo effects, the natural course of osteoarthritis, or regression to the mean. Although the findings suggest potential disease-modifying and anti-inflammatory effects of PRP, these interpretations must be approached with caution. Furthermore, clinical outcomes were not assessed in a blinded fashion, which may have introduced reporting bias. The use of concurrent home-based exercise therapy may also have contributed to the improvements, making it difficult to isolate the specific effect of PRP. In addition, although all patients were instructed in a standardized home exercise program consisting of straight leg raise, hip abduction, and hip adduction training, their adherence was not systematically assessed, which further complicates the interpretation of the observed outcomes. Additionally, while we employed the WORMS system to evaluate cartilage morphology, more sensitive imaging modalities such as T2 mapping or T1ρ were not used [26,27], which may have limited our ability to detect subtle changes in cartilage quality. Finally, the follow-up period was limited to six months, and the long-term sustainability of the observed effects remains uncertain. These limitations underscore the need for future randomized, controlled trials with longer follow-up to validate our preliminary observations.

## 5. Conclusions

This study demonstrated that PRP therapy was associated with improvements in cartilage integrity, as assessed by the WORMS system, specifically in the LFTJ and PFJ, but not in the MFTJ, in patients with varus knee osteoarthritis. These findings suggest that PRP may exert disease-modifying effects primarily in mechanically stable, low-load-bearing compartments. Furthermore, improvement in the PFJ cartilage score was significantly associated with clinical responder status, indicating a potential link between localized structural improvement and symptomatic benefit. A reduction in synovial fluid volume, suggestive of an anti-inflammatory effect, was also observed; however, this change did not correlate with clinical outcomes, implying that synovial volume alone may not be a reliable biomarker of treatment efficacy.

Despite these promising results, the study design using a historical control group remains a major limitation, and definitive conclusions regarding causality cannot be drawn. Although the inclusion of a control group, albeit small in number, strengthens the interpretation of the findings, prospective, randomized controlled trials with long-term follow-up are still required to confirm these preliminary results and to elucidate the mechanisms underlying PRP’s effects on joint structure and symptoms in knee osteoarthritis.

## Figures and Tables

**Table 1:** Demographics of the patients.

Severity of Knee OA (KL Grade 2/3/4)	54/58/109
Sex (male/female)	55/166
Age (years)	68.4 ± 9.5
Body mass index (kg/m^2^)	25.0 ± 4.2

OA: osteoarthritis, KL: Kellgren–Lawrence. Data are presented as n or means ± standard deviations.

**Table 2:** VAS and KOOS before and after PRP therapy.

	Pretreatment	6 Months After PRP	*p*-Value (Wilcoxon Test)
VAS	58.6 ± 25.2	35.0 ± 25.4	**<0.0001**
KOOS—pain	55.0 ± 18.4	67.6 ± 16.9	**<0.0001**
KOOS—symptoms	52.3 ± 19.2	67.1 ± 20.0	**<0.0001**
KOOS—activities of daily living	66.3 ± 18.4	77.3 ± 17.2	**<0.0001**
KOOS—sports and recreation function	29.2 ± 22.0	42.9 ± 28.2	**<0.0001**
KOOS—knee-related quality of life	25.4 ± 17.6	45.5 ± 21.8	**<0.0001**

VAS: visual analog scale, KOOS: Knee Injury and Osteoarthritis Outcome Score, PRP: platelet-rich plasma. Data are presented as means ± standard deviations.

**Table 3:** Magnetic resonance imaging analyses by KL grade in PRP therapy group.

	KL Grade	Pre Treatment	6 Months after PRP	*p*-Value (Wilcoxon Test)
WORMS cartilage score (MFTJ)	KL2	8.7 ± 7.0	8.3 ± 7.5	0.09
	KL3	15.9 ± 7.3	16.1 ± 7.6	0.60
	KL4	23.5 ± 4.6	23.8 ± 4.5	0.12
	Total (KL2~4)	17.9 ± 8.6	18.0 ± 8.9	0.79
WORMS cartilage score (LFTJ)	KL2	0.6 ± 1.4	0.5 ± 1.2	0.17
	KL3	3.7 ± 5.4	3.3 ± 5.0	0.053
	KL4	7.1 ± 7.1	6.7 ± 7.2	**0.009**
	Total (KL2~4)	4.7 ± 6.3	4.3 ± 6.2	**<0.001**
WORMS cartilage score (PFJ)	KL2	3.4 ± 5.1	3.0 ± 4.8	**0.02**
	KL3	7.0 ± 5.5	6.9 ± 5.7	0.65
	KL4	8.8 ± 6.1	8.6 ± 5.7	0.21
	Total (KL2~4)	7.0 ± 6.1	6.8 ± 5.9	**0.048**
WORMS cartilage score (total)	KL2	12.7 ± 11.4	11.8 ± 11.4	0.059
	KL3	26.3 ± 13.5	26.1 ± 13.0	0.28
	KL4	39.6 ± 13.3	39.2 ± 12.8	0.36
	Total (KL2~4)	29.5 ± 16.9	29.0 ± 16.8	**0.021**
WORMS bone marrow abnormality score	KL2	2.3 ± 3.8	2.5 ± 4.3	0.29
	KL3	3.8 ± 4.0	3.6 ± 3.6	0.32
	KL4	6.5 ± 4.9	6.5 ± 5.2	0.94
	Total (KL2~4)	4.8 ± 4.7	4.7 ± 4.9	0.87
Area of synovial fluid (cm^2^)	KL2	1.6 ± 1.3	1.2 ± 1.2	**0.008**
	KL3	2.2 ± 1.7	1.8 ± 1.6	0.17
	KL4	2.3 ± 2.0	2.0 ± 1.7	**0.009**
	Total (KL2~4)	2.1 ± 1.8	1.7 ± 1.6	**<0.001**

WORMS: Whole-organ magnetic resonance imaging score, MFTJ: medial femorotibial joint, LFTJ: lateral femorotibial joint, PFJ: patellofemoral joint. Data are presented as means ± standard deviations.

**Table 4:** Magnetic resonance imaging analyses by KL grade in control groups.

	KL Grade	Baseline	At 6 Months	*p*-Value
WORMS cartilage score (MFTJ)	KL2	3.3 ± 3.3	3.6 ± 3.6	**0.02**
	KL3	14.0 ± 5.1	14.4 ± 5.3	0.50
	KL4	19.2 ± 3.3	19.4 ± 2.6	0.62
	Total (KL2~4)	8.1 ± 7.5	8.4 ± 7.6	0.051
WORMS cartilage score (LFTJ)	KL2	2.4 ± 2.8	2.3 ± 3.1	0.81
	KL3	3.2 ± 1.0	3.3 ± 1.2	0.36
	KL4	11.8 ± 7.5	11.8 ± 8.0	1.00
	Total (KL2~4)	4.1 ± 5.0	4.1 ± 5.2	0.88
WORMS cartilage score (PFJ)	KL2	2.4 ± 4.6	2.7 ± 4.7	0.056
	KL3	4.2 ± 4.8	4.2 ± 5.0	1.00
	KL4	7.4 ± 4.5	7.6 ± 4.7	0.37
	Total (KL2~4)	3.6 ± 4.8	3.8 ± 5.0	0.056
WORMS cartilage score (total)	KL2	8.1 ± 6.1	8.6 ± 6.5	**0.03**
	KL3	21.3 ± 6.5	21.9 ± 6.8	0.36
	KL4	38.4 ± 9.2	38.9 ± 9.3	0.58
	Total (KL2~4)	15.8 ± 13.2	16.3 ± 13.4	**0.01**
WORMS bone marrow abnormality score	KL2	0.2 ± 0.5	0.3 ± 0.8	0.08
	KL3	0.6 ± 0.8	0.5 ± 0.8	0.36
	KL4	0.4 ± 0.5	0.4 ± 0.5	0.99
	Total (KL2~4)	0.3 ± 0.6	0.4 ± 0.7	0.42
Area of synovial fluid (cm^2^)	KL2	1.0 ± 0.9	1.0 ± 0.9	0.62
	KL3	0.7 ± 0.5	0.7 ± 0.7	1.00
	KL4	0.9 ± 0.8	1.9 ± 1.7	0.37
	Total (KL2~4)	0.9 ± 0.8	1.1 ± 1.0	0.08

WORMS: whole-organ magnetic resonance imaging score, MFTJ: medial femorotibial joint, LFTJ: lateral femorotibial joint, PFJ: patellofemoral joint. Data are presented as mean ± standard deviation.

**Table 5:** Correlation between improvement in pain scale score and MRI findings before initiation of PRP therapy.

Magnetic Resonance Imaging Findings	Correlation with Improvement in VAS	
	r (95% CI)	*p*-Value
WORMS cartilage score (MFTJ)	0.21 (0.08–0.34)	**0.001**
WORMS cartilage score (LFTJ)	0.13 (−0.004–0.26)	0.051
WORMS cartilage score (PFJ)	0.16 (0.03–0.29)	**0.01**
WORMS bone marrow abnormality score	0.15 (0.01–0.28)	**0.02**
Area of synovial fluid	0.08 (−0.05–0.21)	0.23

VAS: visual analog scale, CI: confidence interval, WORMS: whole-organ magnetic resonance imaging score, MFTJ: medial femorotibial joint, LFTJ: lateral femorotibial joint, PFJ: patellofemoral joint.

**Table 6:** MRI analyses of responders and non-responders.

Responders	n	Pretreatment			6 Months after PRP			*p*-Value (Wilcoxon Test)
WORMS cartilage score (MFTJ)	**135**	**17.6**	**±**	8.9	17.7	±	9.2	0.72
WORMS cartilage score (LFTJ)	135	4.3	±	5.8	3.9	±	5.6	**<0.001**
WORMS cartilage score (PFJ)	135	7.0	±	6.1	6.6	±	5.8	**0.001**
WORMS cartilage score (total)	135	28.9	±	17.0	28.1	±	16.7	**0.001**
WORMS bone marrow abnormality score	135	4.5	±	4.9	4.6	±	5.0	0.51
Area of synovial fluid (cm^2^)	135	1.9	±	1.4	1.7	±	1.4	0.052
Non-responders	**n**	**Pretreatment**			**6 months after PRP**			***p*-value** **(Wilcoxon test)**
WORMS cartilage score (MFTJ)	86	18.3	±	8.0	18.4	±	8.3	0.91
WORMS cartilage score (LFTJ)	86	5.1	±	7.1	4.9	±	7.1	0.21
WORMS cartilage score (PFJ)	86	7.0	±	6.1	7.1	±	6.1	0.65
WORMS cartilage score (total)	86	30.5	±	16.9	30.5	±	16.9	0.91
WORMS bone marrow abnormality score	86	5.2	±	4.6	5.0	±	4.8	0.33
Area of synovial fluid (cm^2^)	86	2.3	±	2.3	1.8	±	1.8	**<0.001**

**Table 7:** Correlation between improvement in PROMs and improvement in area of synovial fluid.

Improvement in PROMs	Correlation with Improvement in Area of Synovial Fluid (cm^2^)	
	r (95% CI)	*p*-Value
ΔVAS	0.001 (−0.13–0.13)	0.98
ΔKOOS–pain	0.16 (0.03–0.29)	0.017
ΔKOOS—symptom	0.16 (0.04–0.294)	0.012
ΔKOOS—ADL	0.12 (−0.01–0.25)	0.074
ΔKOOS—sports	0.18 (0.05–0.31)	0.007
ΔKOOS—QOL	0.15 (0.02–0.28)	0.024

PROMs: patient-reported outcome measures, CI: confidence interval, VAS: visual analog scale, KOOS: Knee Injury and Osteoarthritis Outcome Score, ADL: activities of daily living, QOL: quality of life.

**Table 8:** Logistic regression analysis of good clinical outcomes (responders) and MRI changes.

Independent Variables (Difference Between Pre- and Post-PRP)	Univariate		Multivariate	
	OR (95% CI)	*p*-Value	OR (95% CI)	*p*-Value
WORMS cartilage score (MFTJ)	1.03 (0.92–1.16)	0.62	1.12 (0.91–1.38)	0.26
WORMS cartilage score (LFTJ)	1.03 (0.86–1.24)	0.75	1.19 (0.87–1.64)	0.27
WORMS cartilage score (PFJ)	1.27 (1.07–1.52)	**0.007**	1.45 (1.09–1.94)	**0.01**
WORMS cartilage score (total)	1.05 (0.97–1.13)	0.22	0.86 (0.69–1.07)	0.17
WORMS bone marrow abnormality score	0.98 (0.88–1.10)	0.79	0.98 (0.88–1.11)	0.84
Area of synovial fluid	0.99 (0.99–1.00)	0.06	0.99 (0.99–1.00)	0.07

Platelet-rich plasma, OR: odds ratio, CI: confidence interval, WORMS: whole-organ magnetic resonance imaging score, MFTJ: medial femorotibial joint, LFTJ: lateral femorotibial joint, PFJ: patellofemoral joint.

## Data Availability

The data that support the findings of this study are available from the corresponding author upon reasonable request.

## Abbreviations

The following abbreviations are used in this manuscript:

PRP	platelet-rich plasma
MRI	magnetic resonance imaging
PROMs	patient-reported outcome measures
WORMS	whole-organ MRI score
MFTJ	medial femorotibial joint
LFTJ	lateral femorotibial joint
PFJ	patellofemoral joints
OA	osteoarthritis
NSAIDs	nonsteroidal anti-inflammatory drugs
KL	Kellgren–Lawrence
BMA	bone marrow abnormality
VAS	visual analog scale
KOOS	Knee Injury and Osteoarthritis Outcome Score
OMERACT	Outcome Measures in Rheumatology responder criteria
OARSI	Osteoarthritis Research Society International
CI	confidence interval
OR	odds ratio
ADL	Activities of daily living
QOL	quality of life
RCT	randomized controlled trial
TNF-α	tumor necrosis factor-α
IL-1β	interleukin-1 beta

## Appendix A

**Table A1:** Supplemental table. Baseline characteristics of responder and non-responder.

	Mean ± SD	*p* Value
Sex (male/female)	R: 28/107 N: 27/59	0.08
KL grade	R: KL2 = 37, KL3 = 38, KL4 = 60 N:KL2 = 17,KL3 = 20, KL4 = 49	0.07
Age (years)	R: 68.9 ± 9.9 N: 67.6 ± 8.9	0.31
Body mass index (kg/m^2^)	R: 25.2 ± 4.5 N: 24.8 ± 3.5	0.80
VAS	R: 62.3 ± 24.0 N: 51.9 ± 26.2	**<0.01**
KOOS—pain	R: 50.3 ± 17.7 N: 62.3 ± 16.9	**<0.01**
KOOS—symptoms	R: 50.0 ± 19.0 N: 55.4 ± 19.1	**0.04**
KOOS—activities of daily living	R: 62.6 ± 19.1 N: 71.9 ± 15.5	**<0.01**
KOOS—sports and recreation function	R: 24.4 ± 20.2 N: 36.1 ± 22.8	**<0.01**
KOOS—knee-related quality of life	R: 21.3 ± 15.7 N: 31.8 ± 18.3	**<0.01**
MTFJ WORMS cartilage score	R: 17.6 ± 8.9 N: 18.3 ± 8.0	0.91
LFTJ WORMS cartilage score	R: 4.3 ± 5.8 N: 5.1 ± 7.1	0.66
PFJ WORMS cartilage score	R: 7.0 ± 6.1 N: 7.0 ± 6.1	0.95
WORMS bone marrow abnormality score	R: 4.5 ± 4.9 N: 5.2 ± 4.6	0.71
Area of synovial fluid (cm^2^)	R: 1.9 ± 1.4 N: 2.3 ± 2.3	0.16
